# Detecting desertification in the ancient oases of southern Morocco

**DOI:** 10.1038/s41598-023-46319-1

**Published:** 2023-11-08

**Authors:** Louise Rayne, Filippo Brandolini, Jen Lavris Makovics, Emily Hayes-Rich, Jackson Levy, Hope Irvine, Lima Assi, Youssef Bokbot

**Affiliations:** 1 School of History, Classics and Archaeology, Newcastle University, Armstrong Building, Newcastle Upon Tyne, NE1 7RU UK; 2 Department of Anthropology, University of New Mexico, Albuquerque, NM 87131-1086 USA; 3 Institut National des Sciences de l’Archéologie et du Patrimoine, Rabat-Institutes (Maroc), Hay Riad, Madinat Al Irfane, Angle Rues 5 et 7, BP 6828, Rabat, Morocco

**Keywords:** Environmental impact, Hydrology, Climate-change adaptation, Scientific data, Climate-change impacts

## Abstract

Understanding what led to desertification in the long-term is crucial for adaptation to climate change and pressures on resources in North Africa, but existing maps do not accurately show the extent of degraded land or the traditional water systems which underpinned cultivation. These products rely on recent vegetation trends and hindcasted statistical data. Desertification which occurred prior to the later twentieth century is poorly represented, if at all. However, large areas of abandoned fields are distinctive in satellite imagery as brightly reflectant and smooth surfaces. We present a new and open-source machine-learning workflow for detecting desertification using satellite data. We used Google Earth Engine and the random forest algorithm to classify five landcover categories including a class representing desertified fields. The input datasets comprised training polygons, a 12-band Sentinel-2 composite and derived tasselled cap components, and a Sentinel-1 VV-polarisation composite. We test our approach for a case study of Skoura oasis in southern Morocco with a resulting accuracy of 74–76% for the desertification class. We used image interpretation and archaeological survey to map the traditional irrigation systems which supply the oasis.

## Introduction

Land degradation is a dedicated sub-indicator of the UN Sustainable Development Goals (SDG 15.3.1)^1^ in recognition of the challenge it poses. The United Nations Convention to Combat Desertification (UNCCD) define land degradation as a binary quantification of the loss of biological and economic productivity^1^. They do not give a clear definition of desertification in their publication addressing the SDG. The exact application of the term degradation is also debated and can sometimes encompass desertification, as well as both natural and human processes^2^. In our paper, we investigate degraded, formerly cultivated land which has been abandoned (due to any factor, including climate change, droughts, groundwater depletion, damage to irrigation infrastructure, socio-political reasons, etc.) and therefore has become desertified in the context of the arid environment of southern Morocco. In North Africa, a complex situation exists with climate change, agricultural intensification, over-abstraction of water, and land and water tenure affecting cultivation. Droughts and floods have increased food insecurity^3^. Projection of increasing desertification is challenging to model given highly variable conditions in drylands, but increased aridity is predicted for North Africa^4^.

**Figure 1:**
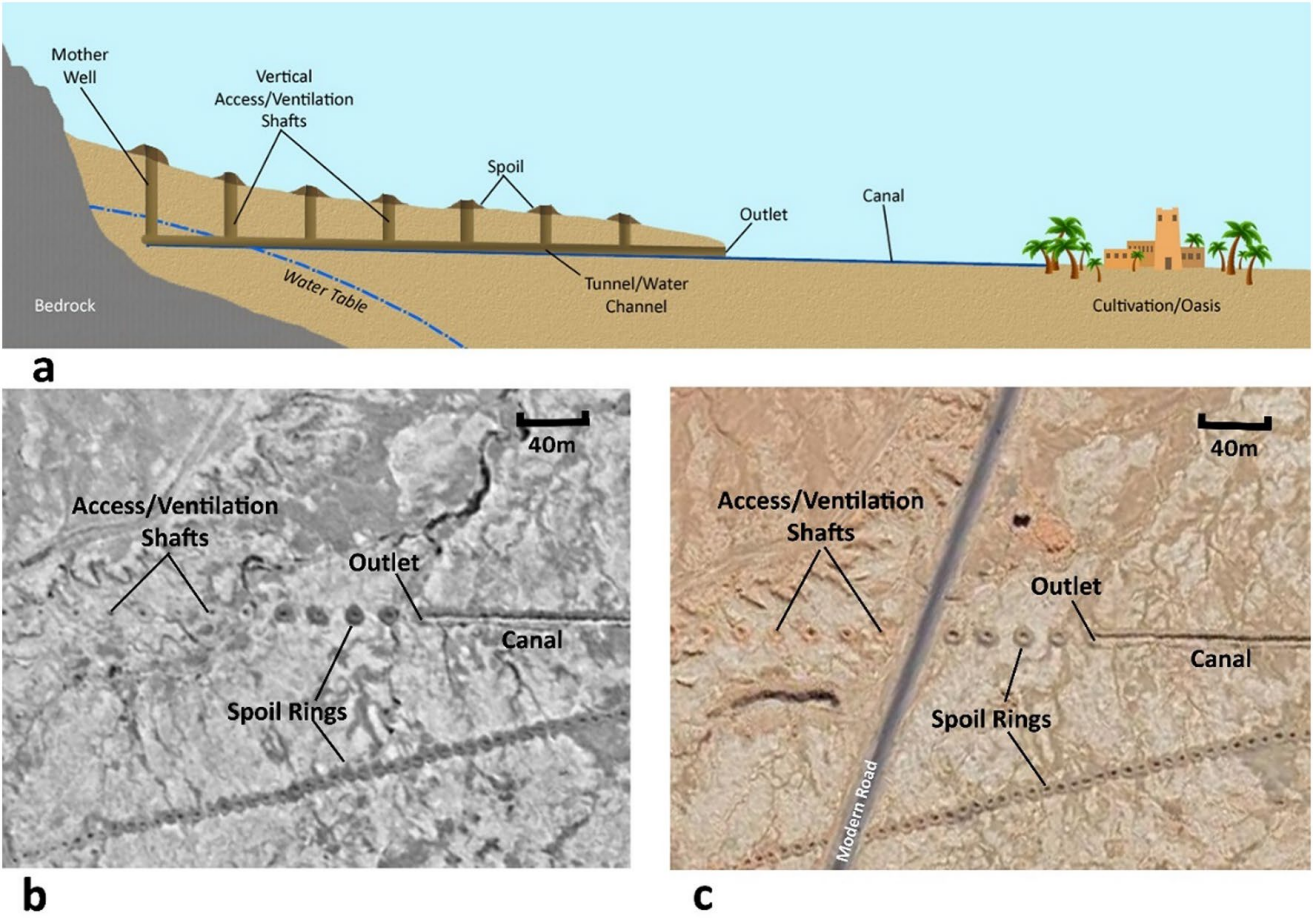
(**a**) Long profile of a typical khettera. (**b**) Satellite image shows khettera shafts and outlet: KH9 1217-100011-F023c, 12th May 1982. (**c**) Satellite image of the same feature: Google Earth Pro map data: 2023 CNES/Airbus, Maxar Technologies https://www.google.com/intl/en_uk/earth/versions/#earth-pro. Image prepared using ArcGIS ArcMap 10.6.1 https://www.esri.com/en-us/home and GIMP 2.10.34 https://www.gimp.org/.

In the Sahara and along its edge, the traditional method of cultivation is the 3-tier oasis system. Palms create a shady, moist microclimate, protecting fruit trees and vegetables and cereals beneath. Oases are facilitated by networks of traditional irrigation systems including earthen canals known as seguia and groundwater-collecting tunnels called khettara (Fig. 1). Khettara are sustainable because they abstract water passively when flow is available^5, 6^. The origins of khettara are complex^7^ but the technology can be found throughout many arid regions. Khettara appear to have reached North Africa from the Middle East via Egypt by the mid-first millennium BC, and Morocco by the eleventh–fourteenth centuries AD^5,7–10^.

These water systems underpin man-made oasis environments which have been developed over thousands of years and are at significant risk from desertification. Arid regions are particularly vulnerable to climate change^4^. In the past, oasis societies dealt with challenges including droughts, floods and regime change, in some cases failing and desertifying, while in others adapting and surviving. Taking an interdisciplinary approach to understanding resilient long-term practices and historic desertification is crucial for future adaptation to climactic stressors. Uncertainty exists around precise quantification of land use changes on the global carbon cycle; accurate mapping of desertified land could improve this^11^.

Conversely, the adaptation literature focuses on oases in their current state as modern landscapes, and maps of the location of desertified cultivation rely on the few years of recent data offered by satellite sensors such as MODIS (2001–2016) or Landsat (e.g. 2000–2019 used by Moussa et al.^12^). The actual extent and potential of degraded land is unknown^2^ and the long-term processes that led to degradation are largely ignored. This seems remiss, given that expansion of modern agricultural production into degraded lands is discussed^2^. Despite this, there are increasing appeals, especially from the archaeological community, to recognise the value and sustainability of indigenous and traditional knowledge regarding cultivation, water management techniques, and the preservation of traditional systems^4, 13–19^.

To address the Saharan degradation lacuna, former cultivation and water management networks should be recorded together with the historical and archaeological evidence to describe the long-term development of traditional landscapes. We have developed an open-source workflow based on machine learning that uses freely-available satellite imagery to detect formerly cultivated areas in arid environments. We use the cloud API Google Earth Engine (GEE) and data from the ESA satellites Sentinel-1 and -2. The code is available in the supplementary material S1 in both JavaScript for GEE and Python for Colab. We also outline our standardised methodology for recording traditional irrigation systems using remote sensing and archaeological and anthropological fieldwork.

### Skoura oasis

**Figure 2:**
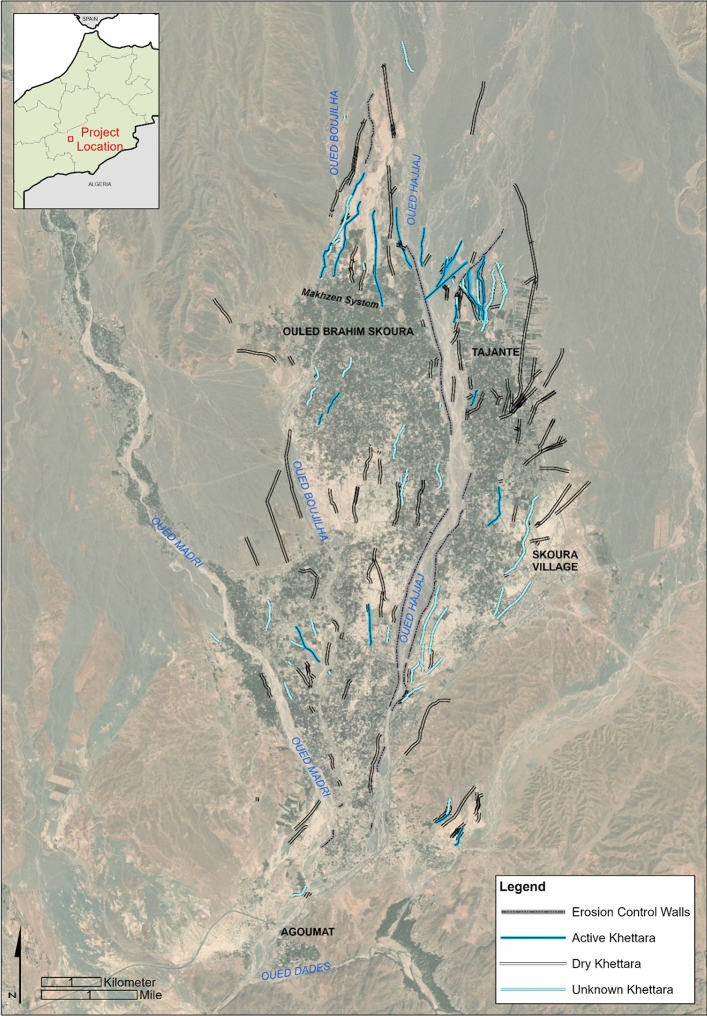
Skoura oasis and khettara irrigation network. Channels recorded as actively flowing or dry at the time of the field visit (Nov-Dec 2022) are labelled accordingly (Fig S1 shows visited khettara and data S1 is the shapefile). Others are labelled according to their appearance on satellite imagery. Areas of desertified soils are light coloured and bright in appearance. Map prepared using ArcGIS ArcMap 10.6.1 https://www.esri.com/en-us/home. Service layer source: ESRI, MAXAR, EARTHSTAR Geographics.

Although this method can be applied to other Saharan oases, our area of interest (AOI) comprises Skoura oasis in southern Morocco (Fig. 2). Skoura is located between and around the confluence of three southward-trending intermittent wadis (Oued el Hajjaj, Oued Boujilha, Oued Madri) that have their origins in the High Atlas Mountains, and flow into the Oued Dadès. Skoura differs from many traditional oases in which groundwater is the primary source of water as historical and modern irrigation practices rely heavily on water from the three wadis combined with groundwater abstraction. Because the wadis typically run from late winter to late spring, Skoura oasis cultivates date palm and fruit trees that require only seasonal irrigation. Thirty-nine percent of farmers do not practice cereal cultivation or market gardening due to a lack of water^20^. Those who do cultivate cereals employ khettara (Fig. 1), the traditional, galleried, underground water abstraction and conveyance system which taps into the water table and which can provide a consistent flow of groundwater.

During low-rainfall periods, Skouran irrigation relies primarily on the khettara (flow of up to 50 l/s^21^). Historically intense drought periods have dried up many khettara, leaving the communities with practically no access to consistent irrigation water. When the High Atlas receives significant precipitation, Skoura will irrigate using canal systems directly fed by the wadis and diverted using small stone-built barrages called agoug^21^. Historically, canals in Skoura were of two classes based upon water supply type. Permanent canals originated at khettara outlets where water was sustained year-round, while inundation canals were fed by wadi seasonal flows. Today canals are also supplied by pump wells, with some supplemented by the few remaining functional khettara which now may only produce water intermittently, and by wadi offtakes. Although the traditional oasis irrigation system survives to some degree, desertified areas exist throughout the oasis.

### Mapping traditional cultivation and land degradation

Earth observation has become a crucial tool for assessing land degradation as well as mapping paleo-landscapes^22, 23^ and threats to heritage sites and landscapes^24–27^. Orengo et al. pioneered the use of multi-sensor classification in their work to detect archaeological mound settlements in Pakistan^28^. They also used Google Earth Engine, with Sentinel-2 bands and VV and VH Sentinel-1 polarisations as inputs into a Random Forest classifier. They found many previously unmapped settlements which they linked to changes in the extent of the desert.

Optical satellite imagery can be processed using band ratios to clearly delineate vegetated areas and to produce time-series of changes. SAR (Synthetic Aperture Radar) satellite data can also be applied in arid and semi-arid zones to map agricultural areas and changes in their extents. Machine-learning is increasingly used for mapping landcover. The random forest algorithm is an accurate and powerful approach^29, 30^ and there are a number of examples of applying it in Africa, e.g.^31–33^. Random forest generates ‘trees’ which vote for the most popular class while preventing overfitting, with random inputs taken at each node to grow the trees^29^.

Training data for machine-learning can be derived from existing datasets, but often has to be generated manually by expert image analysts. The combination of multiple datasets into machine-learning algorithms facilitates more nuanced automated mapping, but as Stewart et al.^34^ found, additional inputs do not always enhance the model. Schulz et al.^31^ combined Sentinel-1 (SAR) and Sentinel-2 (optical) to classify land use in the Sahel with an overall accuracy of 72% but were unable to detect traditional rainwater-fed planting pits.

There is unrealised potential to utilise Earth observation for mapping long-term desertification in oasis environments, recognising areas of traditional cultivation as key elements of an oasis’ cultural heritage. There are many areas of historical desertification in the Sahara, which because they were last cultivated prior to the Landsat era (1970s-80s onwards) and before comprehensive statistics were gathered, tend not to get included in land degradation maps. The existing global landcover datasets for desertified land in Africa fall short since it is incorporated into a bare soil class; therefore, their accuracy and interoperability is relatively low^31^.

Published maps of desertification focus on recent periods (post-1980), are generally dependent on proxies, NDVI time-series, and existing land-cover products. They are low-resolution and inaccurate. An approach based on the convergence of statistical data is favoured by UNCCD^1^. Some of these global products are open source. The Trends.Earth dataset calculates a land degradation layer from the outputs of three algorithms; productivity, landcover and soil carbon with resolution of 250 m−8 km^35^. Productivity is generated using MODIS NDVI time-series, comparing baseline and recent data. ESA CCI landcover (from MERIS) is used to identify changed pixels. Soil-organic carbon is calculated using the SoilGrids dataset and coefficients selected by UNCCD. Areas identified as degrading by any one of the inputs are labelled as degraded. The World Atlas of Desertification^36^ also integrates landcover mapping with a measure of productivity, and Moussa et al.^12^ measure degradation in southern Morocco in higher resolution through calculating land productivity, but these datasets are not downloadable or open-source.

Products that model historical cultivation extents, which can be used to infer trajectories of degradation, are low resolution and based on modelled and simulated data and multi-criteria evaluation. Provided as part of the SAGE database^37^, Ramankutty and Foley^38^ gathered historical cropland inventory data and calculated the ratio of past croplands to a 1992 land cover croplands dataset to hindcast cultivated areas from 1700 to 1992. However, this product is of limited use because of its low resolution, the assumption that spatial patterns in 1992 and the past were identical, and the very limited estimates for Africa that were used as proxies for historical data. Similarly, the HYDE database uses the earliest FAO data (1960) and land use estimates from the ESA landcover data, amongst input data derived from other models^11^.

The Tasselled Cap algorithm is a well-established approach in remote sensing which transforms satellite imagery to define the brightest, wettest, and greenest components. Significant information is captured by combinations of image bands transformed by applying matrices of coefficients^39^. These have been derived for sensors including Sentinel-2^40^ and can be fixed for each sensor^39^. The algorithm was originally developed in order to map and assess crop development using Landsat data^41^.

Lamqadam et al‘s^42^ applied the Tasselled Cap algorithm to a single Sentinel-2 image covering the Draa, a perennial river which is supplied by the Mansour Eddahi Dam near Ourzazate, fed similarly to Skoura oasis by snowmelt and precipitation from the Atlas Mountains. They utilised unsupervised classification applied to a ratio of the wetness and brightness component bands to assign degrees of desertification with an accuracy of 93%. They found that bright soil corresponds with poor, dry, soil of organic matter. El Hairchi et al.^43^ applied Tasselled Cap to a forested region of the Middle Atlas but found a ratio of greenness-and brightness components most relevant, achieving a classification accuracy of 88%.

Lamqadam et al.^42^ study is limited because it relies on a single Sentinel-2 image and unsupervised classification of only 1 product (wetness-brightness) and does not consider long-term and historical processes of desertification. Both Lamqadem^42^ and El Hairchi’s^43^ studies do not clearly describe the difference between never-cultivated steppe and areas of historical desertification. However, Gibbs and Salmon^2^ recognise that satellite-based land productivity methods cannot sufficiently capture degraded land, especially when the desertification happened long ago. They call for new approaches using remote sensing and field inventories to classify degraded lands. The present study addresses this by taking a multi-sensor, machine-learning remote sensing approach combined with archaeological survey to map abandoned croplands and their irrigation systems.

## Methods

### Image interpretation

To identify extant traditional hydraulic management features in and around Skoura Oasis we conducted a systematic visual scan of the imagery available through Google Earth Pro (GE) and created a record of each digitized feature (see supplemental data S1 for shapefile and GeoJSON of mapped and visited khettara). Using GE has key benefits: the platform is free and has relatively user-friendly digitization tools, the Keyhole-Markup-Language (KML) data may be easily exported to GIS shapefiles (.shp) and vice versa, the imagery is very-high-resolution (VHR), and recent imagery is archived in the GE application, offering a fairly-regular image time-series^44, 45^. This facilitates the identification of features that may have been modified, destroyed, or obscured by cloud or shadow.

To ensure all areas were methodically examined, the AOI was overlain with the Military Grid Reference System (MGRS) 10 km Square Identifier shapefile developed by the US military (and used as standard by NATO, see^46^) that is based on the Universal Transverse Mercator (UTM) system^47^. The 10 km^2^ blocks were visually scanned in north–south or east–west transects at an eye-height ranging from 300 to 900 m, with spot checks from 70 to 300 m. Hydraulic management features were digitised using the point, line, and polygon tools (see supplemental materials for image interpretation key, Table S1). A unique identification number was assigned to each feature to ensure that characteristic data captured from historic satellite imagery was matched to the proper feature. Khettara were digitised as segments, with branches digitised as distinct entities from main courses with a unique identification number; this was to facilitate the analysis of construction/modification dates for each segment. Digitization was supervised and undertaken by Lavris Makovics and supplemented by undergraduate students Irvine and Assi during a summer research project in July 2022, for which one week was spent on training students to an operational standard, and one week on digitisation.

Digitized GE.kml files were exported to QGIS, converted to shapefiles (.shp) and overlain on orthorectified declassified Cold War satellite panchromatic imagery from the KH9 HEXAGON program (1971–1982) which achieved resolutions of 0.6–1.2 m. The image shown in this paper was from 12th May 1982 and was orthorectified with an RMSE of 3.21 m. The workflow used to process the KH9 imagery, and the information that it reveals, will be discussed in detail in a further publication. Once rectified, this relatively inexpensive VHR imagery facilitated the re-location of features identified on GE imagery. This permitted an initial assessment of feature and landscape change. It allowed for the establishment of potential earliest and/or latest dates for each feature. Imagery from the earliest KH9 mission (1201) in 1971 and final mission (1217) in 1982 were utilized in this first evaluation. Remote sensing of the KH9 imagery was conducted similarly to the GE methodology to identify additional features not identified from GE. The historic KH9 imagery also facilitated a visual assessment of the growth or retraction of the cultivation and settlements. An assessment was made from the recent imagery considering whether a water feature appeared dry, potentially active, or if this was indeterminate. All features were logged into an Excel spreadsheet. This allowed for easy collation with field data and transfer into GIS for analysis.

### Automated desertification detection

As discussed above, existing methods for assessing landcover degradation are inadequate for identifying the extent of historical desertification. Although oases which went out of cultivation prior to the 1970s/80s are not detectable using vegetation-index time-series, in satellite imagery the bare soils of former fields are distinctive from other bare desert soil classes due to a particularly smooth, bright appearance, colour, and texture.

To ensure ongoing replicability across large regions, we took an open-source approach using GEE. We produced a bespoke script in the JavaScript API via the Earth Engine Code Editor to map landcover including a class for desertification (see supplementary information S1 for open-source code). The GEE platform combines a multi-petabyte catalogue of geospatial datasets and provides a library of algorithms and a powerful application programming interface (API)^48^. Additionally, we also provide a version for the Python API in Google Colab, a serverless Jupyter notebook^49^ computational environment designed for interactive development in Python^50^ using Google Cloud^51^. Colab allows easy sharing and version control. For accessing the Sentinel-2 satellite data, we utilised the Python module geemap in Colab^52^. Although the native GEE Python API has limited functionality in visualising results, the geemap Python module was specifically created to overcome this shortcoming. 

**Figure 3:**
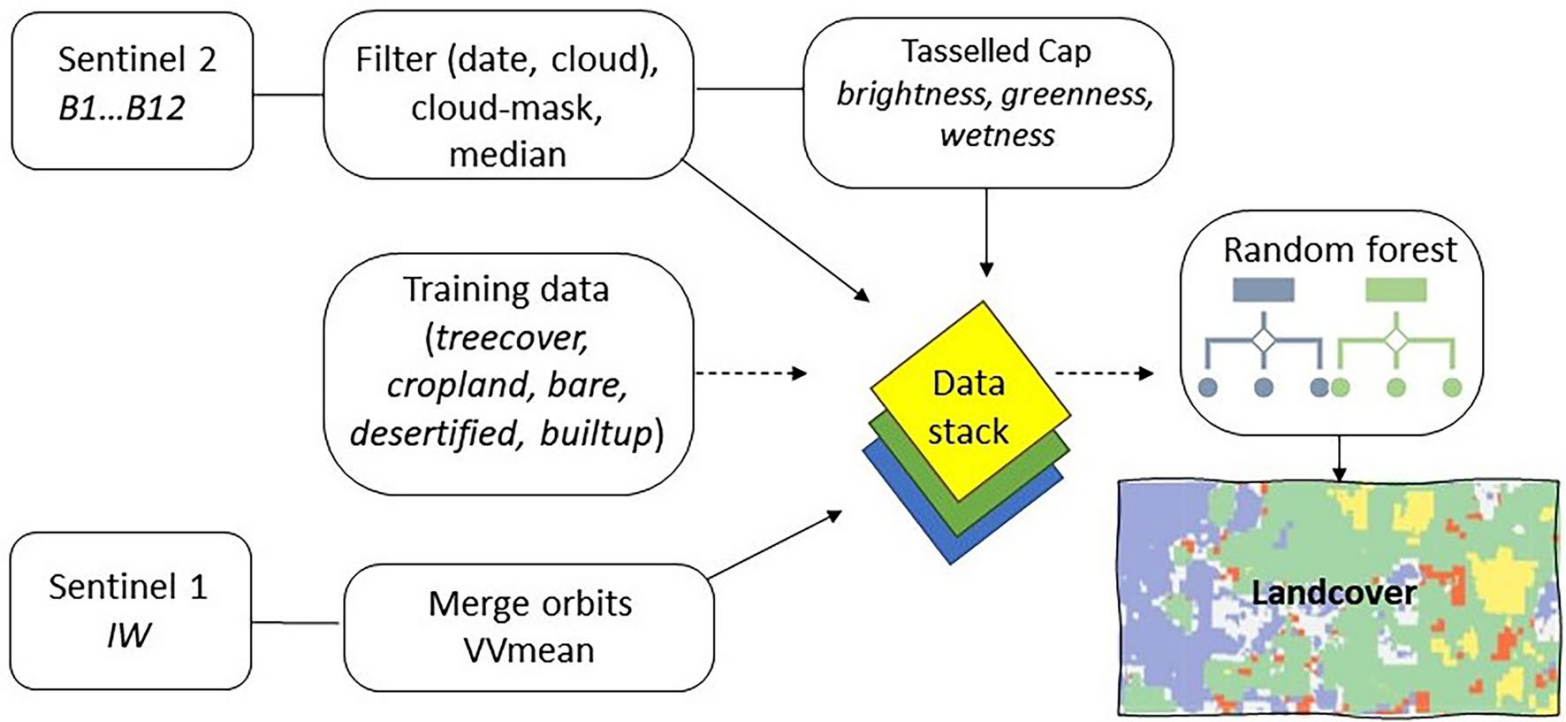
Google Earth Engine Desertification Detection workflow https://developers.google.com/earth-engine.

We took a multi-sensor approach using data from ESA’s open-source Copernicus programme (Fig. 3 indicates workflow). Sentinel-2 provides optical satellite imagery with a resolutions of 10, 20 and 60 m across 13 spectral bands between 0.44 and 2 µm. Sentinel-1 captures SAR data in up to 4 polarization bands of 10 m resolution. These datasets were selected because Sentinel-2 can be used to detect distinct spectral signatures, especially vegetated and impervious materials and the bright soil of formerly cultivated fields. Sentinel-1 is useful for analysing surface roughness, including the separation of different types of bare soil. The polarimetric channels of Sentinel-1 detect scattering types representing landcover^53^. The VV band channel in particular shows surface materials including bare ground. Stewart et al.^34, 54^ found this channel to be the most useful polarisation for detecting roads in similar desert environments in Tunisia and Sinai, while they found that the VH channel decreased accuracy, possibly due to increase in speckle.

The Sentinel-2 Level 1C collection is processed to top of atmosphere reflectance, which proved adequate for our analysis and is in keeping with the products used to generate the coefficients of a key input^40^. The collection was filtered to the study area and a date range of 2021-06-01 to 2021-8-31. This represents the drier months in southern Morocco where there is likely to be less influence from seasonal, non-cultivated vegetation, and the summer immediately preceding our field seasons (November–December 2021 and 2022). Further filters were applied to remove the effects of clouds, and a 13-band median composite was generated. We then used the Tasselled Cap technique. The Sentinel-2 composite was first flattened into an array, a set of coefficients was applied, and the result separated into the components of brightness, wetness, and greenness. Although alternative coefficients have been published^55^, we used the Sentinel-2 Tasselled Cap coefficients presented by Nedkov^40^, following the methodology of Lamqadem et al.^42^ who successfully mapped desertification in a neighboring oasis (Table 1).

**Table 1:** Sentinel-2 bands used and tasselled cap coefficients^40^.

Band	Brightness	Greenness	Wetness
B1 aerosols	0.0356	− 0.0635	0.0649
B2 blue	0.0822	− 0.1128	0.1363
B3 green	0.1360	− 0.1680	0.2802
B4 red	0.2611	− 0.3480	0.3072
B5 red-edge 1	0.2964	− 0.3303	0.5288
B6 red-edge 2	0.3338	0.0852	0.1379
B7 red-edge 3	0.3877	0.3302	− 0.0001
B8 NIR	0.3895	0.3165	− 0.0807
B9 water vapor	0.949	0.0467	− 0.0302
B10 cirrus	0.0009	− 0.0009	0.0003
B11 SWIR 1	0.3882	− 0.4578	− 0.4064
B12 SWIR 2	0.1366	− 0.4064	− 0.5602
B8a red-edge 4	0.4750	0.3625	− 0.1389

The Sentinel-1 collection comprising Ground Range Detected (GRD) scenes was filtered by the same dates. This collection is provided in the GEE catalogue pre-processed for thermal noise removal, radiometric calibration, and terrain collection. Data in the IW (Interferometric Wide Swath) mode was selected. The data from both ascending and descending orbits was used and a mean composite of the VV band (single co-polarization, vertical transmit/vertical receive) retained. The VV polarisation seems to have slightly clearer separation between desertified fields and bare ground for our study than the VH, on a visual examination.

Training data for five landcover classes (desertified, builtup, bare, treecover, cropland) was produced (Table 2) for Skoura. To adapt the algorithm most effectively for other areas, new, localised training data should be collected. Like Schulz et al.^31^, we found best heterogeneity of training samples by digitising and labelling Google Earth imagery. Because desertified areas are poorly realised in existing landcover products, we needed to create our own training data rather than using an existing open-source dataset, unlike the OSM data Stewart et al.^34^ take advantage of to detect roads.

**Table 2:** Training data used for landcover mapping.

Training data polygons	
Treecover	30
Cropland	30
Bare	35
Desertified	41
Builtup	38

**Figure 4:**
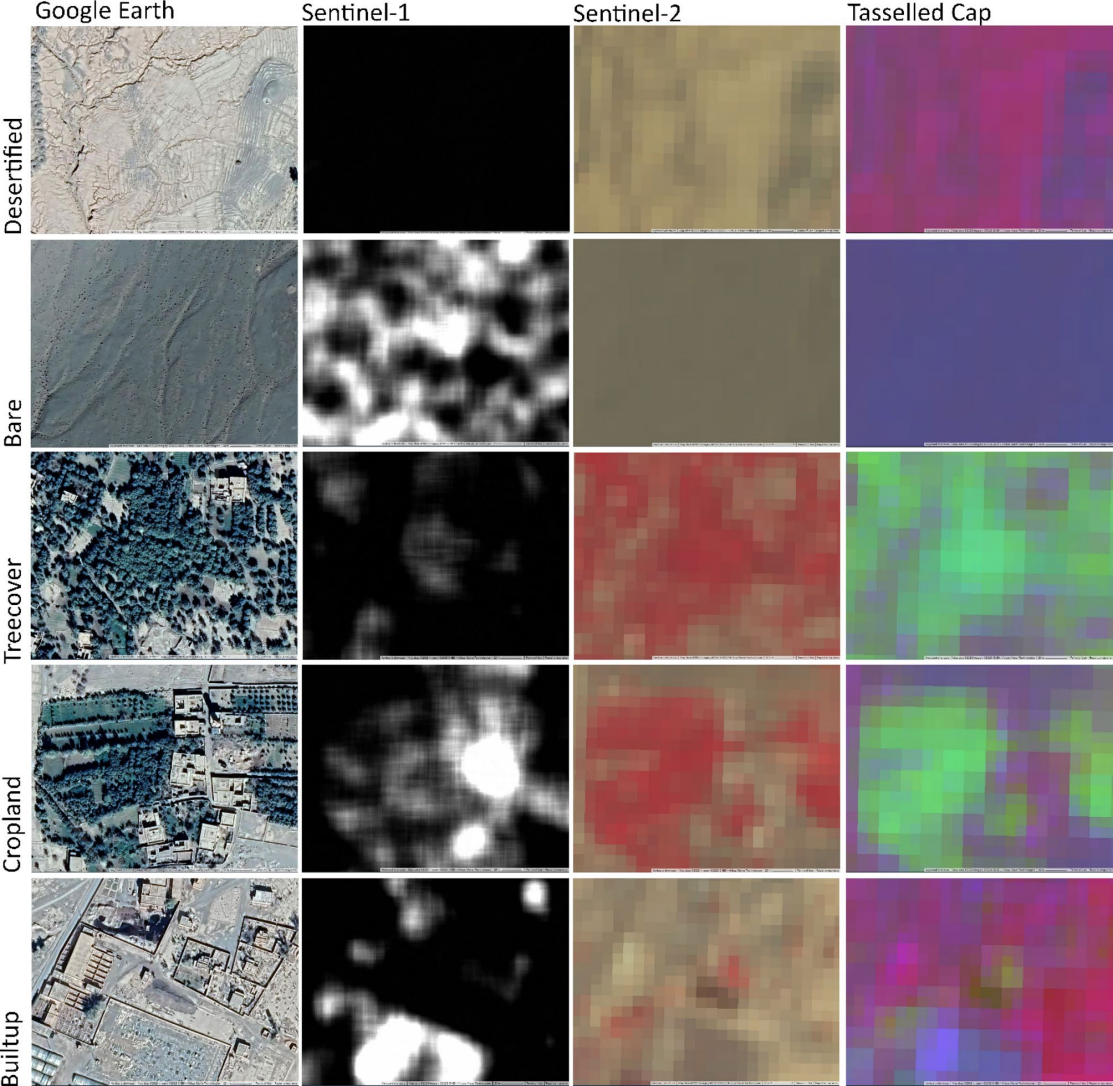
Example of landcover types represented in each input into the data stack. The VV band of Sentinel-1 is used. The Sentinel-2 image is a false colour composite (near-infrared, red, green) and the Tasselled Cap is a composite of the brightness, greenness and wetness components derived from Sentinel-2. The Sentinel data was accessed via Google Earth Engine https://developers.google.com/earth-engine : Copernicus Sentinel data 2021 and contains modified Copernicus Sentinel data 2021. High resolution images were obtained via Google Earth Pro map data: Google, ©2023 CNES/Airbus, Maxar Technologies https://www.google.com/intl/en_uk/earth/versions/#earth-pro. Images prepared using InkScape 1.2 https://inkscape.org/.

The inputs were then merged into one data-stack of 17 bands (Fig. 4). We applied the random forest algorithm to the data stack (13 Sentinel-2 bands, 1 Sentinel-1 polarisation band, and the 3 Tasselled Cap components). Google Earth Engine applies nearest neighbour interpolation to the different spatial resolution bands by default, to the specified scale (in this case 10 m). We trained the Random Forest algorithm using the polygon samples for the 5 assigned classes. Points were then sampled from each polygon while retaining the class labels. The model was restricted to use ten decision trees with unlimited nodes.

### Validation

Validation data was created by sampling 100 randomly generated points for each landcover class (n = 500), mirroring the methods used by Schulz et al.^31^. In the Sahara, the predominant land cover class is bare soil, so sampling across the entire landscape would bias checking to this class. The results of the classification were appended to the points and then each was manually labelled based on the Sentinel-2 and Google Earth images of the same date. An error matrix was constructed to show the accuracy of the sampled results^56^.

### Field survey

**Figure 5:**
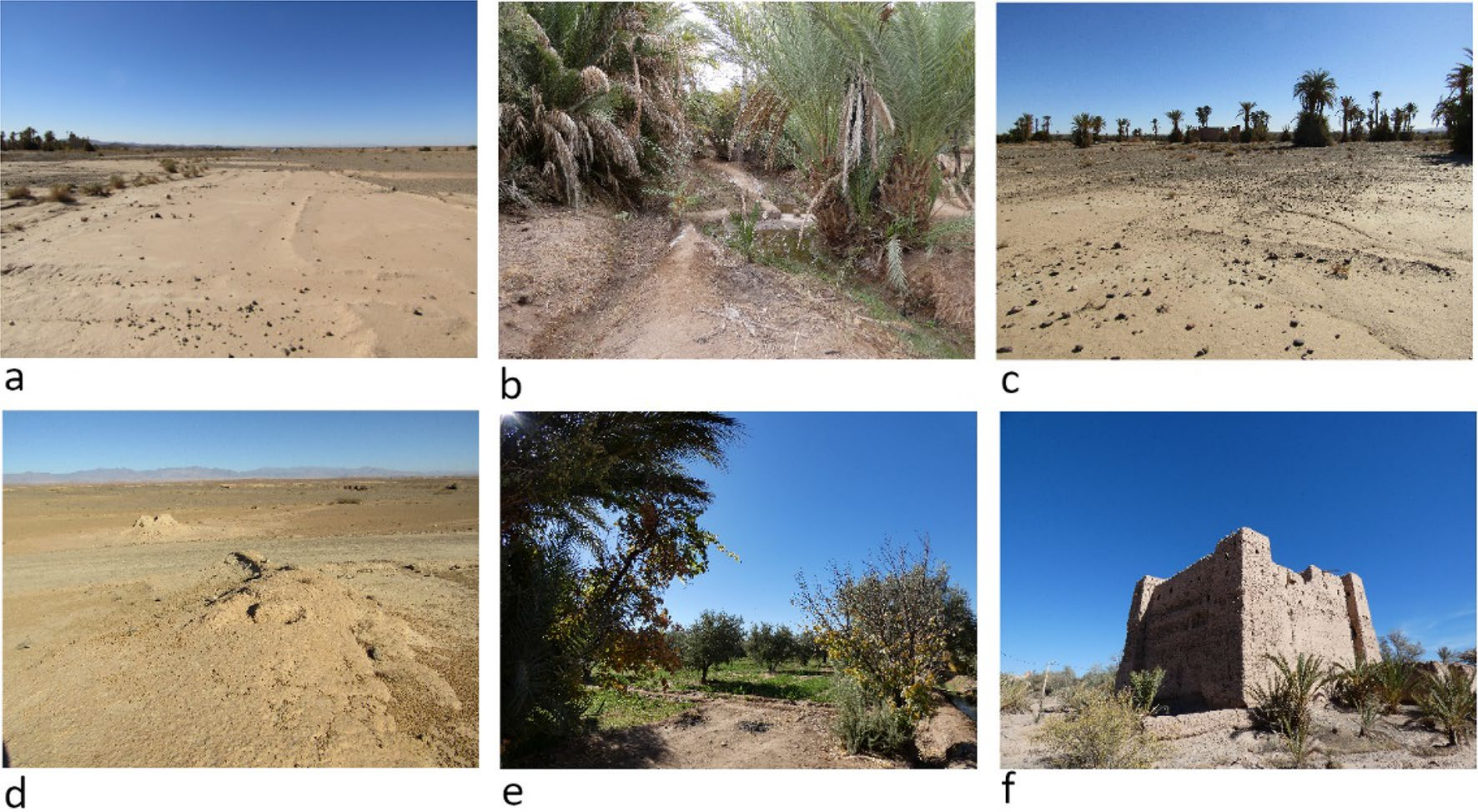
Land cover materials visited and photographed by Lavris Makovics and Rayne November–December 2022. (**a**) Desertified fields with a typical smooth and bright appearance. (**b**) Dense palm cultivation. (**c**) Desertified fields with some surviving palms. (**d**) Natural material. (**e**) Typical 3-part oasis with palms, fruit trees and crops. (**f**) Mudbrick and pisé kasbah. Image prepared using GIMP 2.10.34 https://www.gimp.org/.

Limited time for ground-truthing in the Skoura area necessitated that ease of access and areas with multiple features were selected for focussed pedestrian survey (reconnaissance in November–December 2021, survey in November–December 2022). Fifty-two of 268 digitised khettara segments were visited (19%). We used the QField mobile application^57^ loaded with all digitised khettara, canals and the classification results. This application facilitated swift targeting and navigation to features for ground truthing image interpretation results and validation of the algorithm. Each visited feature was recorded with photographs and points were logged with a Garmin GPSMap 62S. Data regarding each feature were recorded in field notebooks; this included observations on the form and current use/disuse, its environmental and geographic location, current condition, any potential threats to the feature or surrounding area, and the land use category (examples in Fig. 5a–f, and see supplemental material S1). Locations were also compared to the output result of the desertification detection. Whether a channel was dry or active was recorded based on water flowing at the outlets at the time of our survey (see Fig. 1); both the historic and recent satellite imagery shows irrigation is however much more dynamic. Land cover which was not easily distinguishable on the imagery was verified, for example a spread of reflectant natural material was sometimes misclassified as desertified (see Fig. 5d).

Data collected for each khettara included the following:

- An assessment of the khettara type (main line or branch to a main line), and the segment’s relationship to other khettara—including a consideration of potential systems comprised of several khettara lines.
- Current segment length in metres (from digitisation).
- Previous length in metres (from both GE and historic imagery).
- Related features (khettara outlets, reservoirs, canals, etc.).
- The community/communities the khettara and associated canals served.
- The earliest/latest image on which the khettara was observed, including an assessment of historic declassified KH-9 Hexagon spy satellite imagery.
- A determination if the feature is active or relict and evidence for/against.
- Evidence of alteration.
- Evidence of recent (within past 20 years) maintenance.
- Condition
- An estimation (%) of the certainty of identification and interpretation.

Discussions with oasis community members were undertaken as part of a wider project on Moroccan khettara by Hayes-Rich and Levy^10^. Discussions sought to understand the life-history of the khettara, including construction, use, and abandonment, as well as current beliefs and understandings of khettara as a modern water management tool. This research methodology situated the community’s knowledge at the forefront of the survey. During these discussions, community members were encouraged to indicate how this research would be most beneficial for their community. Often, people requested maps of their khettara system, informational brochures, or detailed reports which were provided after fieldwork.

### Ethics declarations

All interviews and local communication were in compliance with the Institutional Review Board regulations through the University of New Mexico. Informed consent of all participants engaging in formal interviews were obtained by Hayes-Rich.

## Results

### Validation of automated desertification detection

The algorithm was reasonably successful at identifying areas of desertification, with a user’s accuracy of 74% and producer’s accuracy of 76% (Table 3). The successful classification of this class was mainly due to the homogeneous appearance of this material in the Sentinel-1 data compared to the other landcover types (Fig. 4). Similarly in the Tasselled Cap composite it is distinct from other types of bare soil (Fig. 4).

**Table 3:** Error matrix. The overall accuracy is 69%.

Classification	Reference					
	Treecover	Cropland	Bare	Desertified	Builtup	Total
Treecover	96	2	0	1	1	100
Cropland	55	23	7	9	6	100
Bare	2	0	94	2	2	100
Desertified	0	0	22	74	4	100
Builtup	6	4	22	11	57	100
TOTAL	159	29	145	97	70	500

Producer's accuracy		User's accuracy				
Treecover	0.6	Treecover	0.96			
Cropland	0.79	Cropland	0.23			
Bare	0.65	Bare	0.94			
Desertified	0.76	Desertified	0.74			
Builtup	0.81	Builtup	0.57			

The producer’s accuracy of 76% for the desertification class shows that some pixels which should have been classified as desertified were misclassified, predominantly as bare; differences between dry desert soils and dry former field soils can be subtle especially due to confusion caused by patches of gypsum substrate (Fig. 5d). Desertified pixels were also misclassified as builtup. This may be because many oasis buildings are constructed from mudbricks or pisé blocks and are therefore spectrally similar to dry fields (Fig. 5f). In general, the builtup class was not as accurate. Builtup materials in arid areas are notoriously difficult to classify^32^. Pixels incorrectly identified as cropland, rather than as desertified, may be due to mixed landcover of surviving palms in amongst otherwise abandoned fields (Fig. 5c).

**Figure 6:**
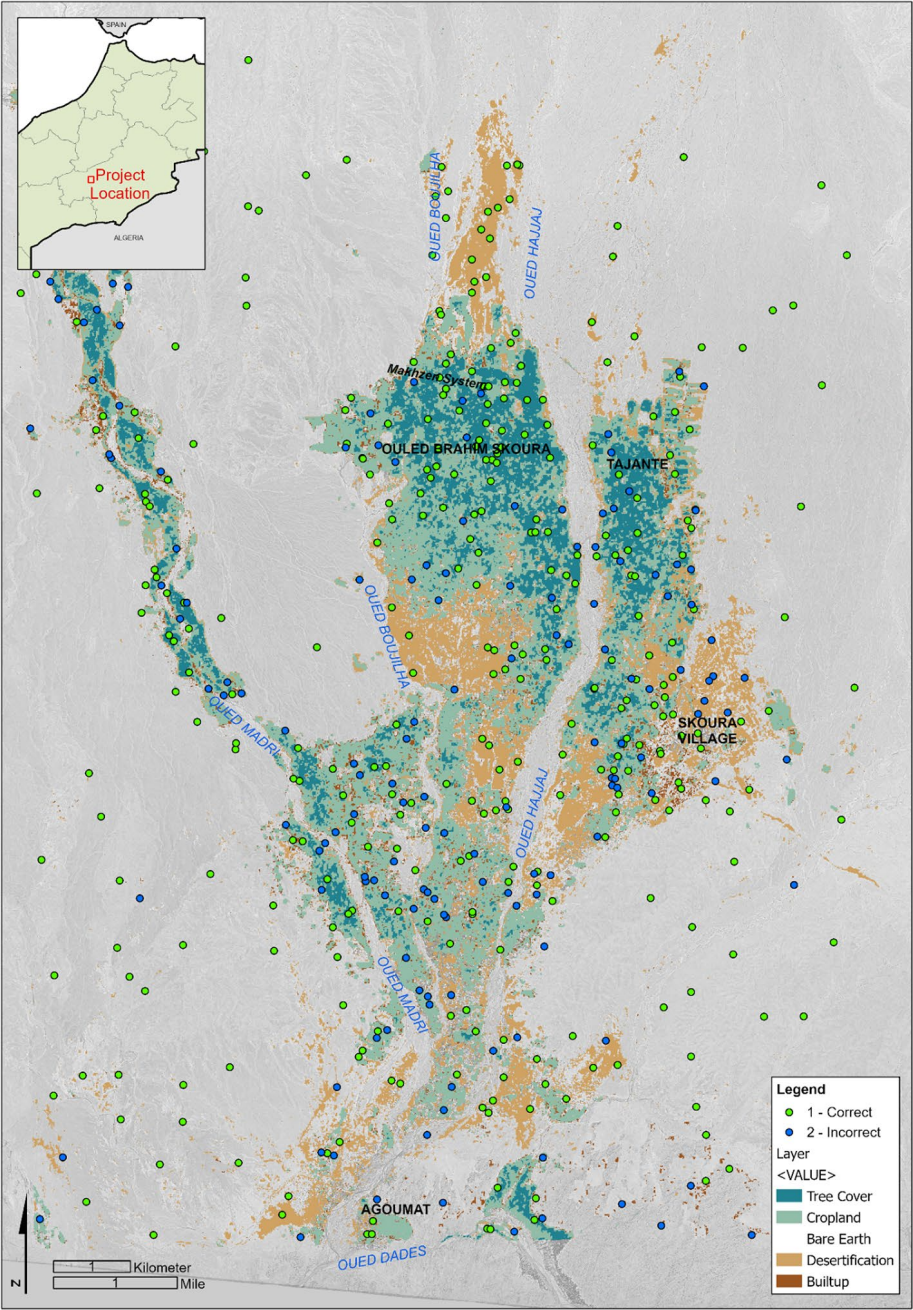
Classification result with proportion of sample points correctly classified. The Sentinel data was accessed via Google Earth Engine https://developers.google.com/earth-engine: contains modified Copernicus Sentinel data 2021. Basemap is KH9 1217-100011-F023c, 12th May 1982. Classification generated using Google Earth Engine and map prepared using ArcGIS ArcMap 10.6.1 https://www.esri.com/en-us/home. Service layer source: ESRI, EARTHSTAR Geographics.

The validation exercise demonstrates varying degrees of accuracy across the different classes with an overall accuracy of 69% (Fig. 6). We found that there was a lack of separation between the two vegetation classes, which led to low accuracy scores for both. The treecover class comprises palms, fruit trees, and scrubby trees such as tamarisk, while the cropland class consists of ground-based crops. In traditional oasis cultivation, such as that of Skoura, cultivation tends to involve small fields with a mixture of palms, fruit trees, and ground-level crops (Figs. 4, 5e). Consequently, a 10 m pixel containing ground-level crops, also likely contains trees/palms (see Fig. 4). Given that it is not possible to consistently separate these, the cropland and treecover classes should be interpreted together.

The Tasselled Cap is a key input. Future work to test the Tasselled Cap algorithm for detecting desertification could comprise a more comprehensive comparison of different sets of coefficients and processing levels. We used the TOA (top of atmosphere) Sentinel-2 product in order to be consistent with the chosen coefficients^40^—there are still only limited published coefficients derived from surface reflectance^58^. Results produced using both TOA and BOA (bottom of atmosphere) were initially compared against the imagery used for validation, but neither appeared to be more accurate than the other. The 2022 field survey included comparison of visited locations with the desertification detection output derived using the TOA data, so in the interests of consistency we used that product for the full analysis presented in this paper.

### The irrigation network around Skoura

Khettara systems are comprised of three distinct elements (Fig. 1); the khettara itself with its subsurface channel and vertical access shafts, an outlet where water comes to the surface, and a main canal, which then carries the water toward use areas through a system of subsequent branch and distributary canals. To ensure water availability, reservoirs may be constructed anywhere along the canal lines; though traditionally they are sited near the outlet. The canal network within Skoura oasis is labyrinthine, and our fieldwork demonstrated that many canals in actively cultivated areas are not easily or fully digitizable solely with remote sensing. The following analysis was conducted for the khettara found in and around Skoura Oasis.

**Figure 7:**
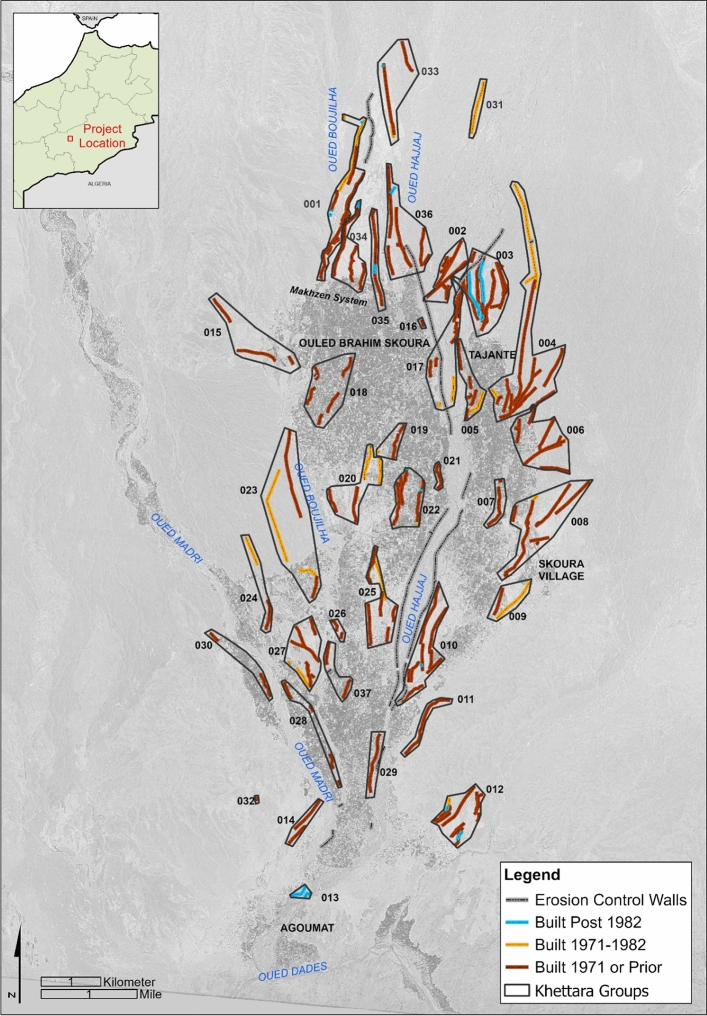
Khettara Groups and chronology of khettara construction in Skoura oasis based on analysis of historic KH9 satellite imagery. Note desertification around Groups 10 and 20. Basemap is KH9 1217-100011-F023c, 12th May 1982. Classification generated using Google Earth Engine and map prepared using ArcGIS ArcMap 10.6.1. https://www.esri.com/en-us/home. Service layer source: ESRI, EARTHSTAR Geographics.

Thirty-seven distinct groupings of the segments were identified from the digitisation and field visit efforts (Fig. 7). Some, such as Groups 31 and 33, historically fed agricultural areas that are now relict, while others, such as Group 1 contain khettara that are still producing water today. Group boundaries were digitised based upon the locations of the khettara, the direction of khettara and canal flow, and the potential communities that the systems fed based on GE (2023) and OpenStreetMap (OSM)^59^. The khettara groups were located strategically around and within the oasis, with the highest concentration of khettara in the nine groups (Groups 1–5, 17, 34–36) located immediately to the north and northeast of the currently green oasis (Fig. 7).

**Figure 8:**
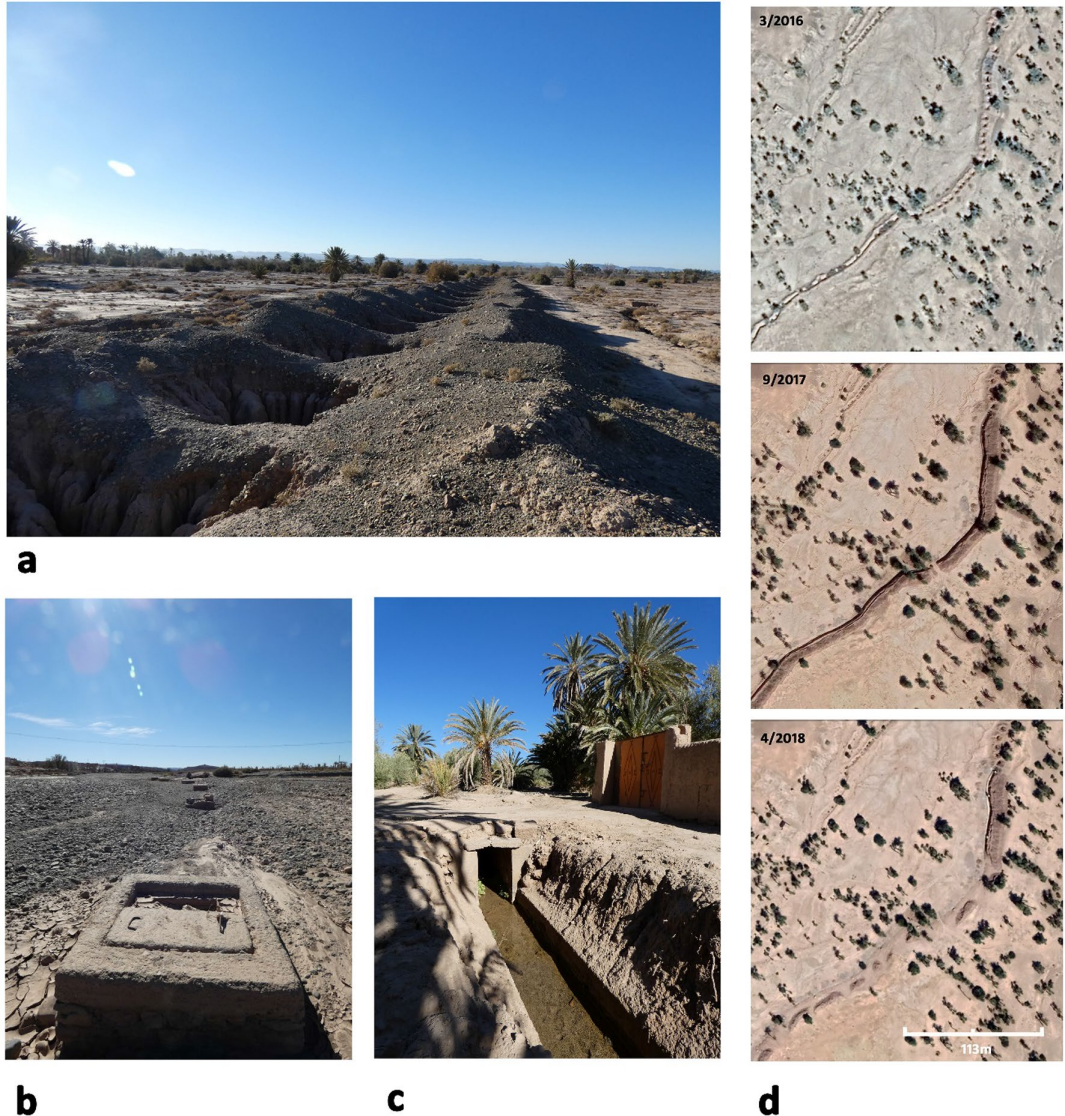
(**a**) Line of khettara shafts on the ground. (**b**) Line of shaft revitalised with concrete. (**c**) Water flowing from outlet lined with concrete. (**d**) Imagery showing stages of revitalisation of khettera. Photographs taken by Makovics and Rayne November–December 2022. Google Earth Pro map data: 2016 CNES/Maxar Technologies, 2017 MAXAR Technologies, 2018 MAXAR Technologies https://www.google.com/intl/en_uk/earth/versions/#earth-pro. Image prepared using GIMP 2.10.34 https://www.gimp.org/.

Twenty-five percent of the 52 visited khettara segments were flowing at the time of the field visit, 46% were dry, and the status of a further 29% was unclear. Two hundred and sixty-eight total khettara segments (including the 52 visited) were digitised within and around Skoura Oasis. Of those 268, 29 (11%) were deemed as actively producing some water, while 171 (64%) were noted as dry/inactive, and 68 (25%) as of unknown status. This was assessed by a combination of ground-truthing and remote sensing of the 52 visited segments (November–December 2022) and solely remote sensing for the remaining 216 segments. The presence of identifiable khettara outlets and associated canals (especially those running to actively cultivated fields), and evidence of recent (≤ 15 years) maintenance or system upgrading on satellite imagery shows a dynamic situation with systems sometimes dry (including at the time of our visit) but potentially intermittently functioning in recent years. Evidence of khettara maintenance includes silt cleanout and re-excavation and concreting of underground systems. Changes in size or configuration of the spoil heaped around each vertical shaft can indicate regular clearance of built-up sediment from within the underground channel (Figs. 1, 8). Revitalisation of khettara may be observed in satellite imagery as lines of former earthen shafts that have been concreted and sometimes capped. Some in-progress line revitalisation work has been captured in recent satellite imagery (Fig. 8).

## Discussion

Our high-resolution, multi-sensor, machine-learning approach mapped large areas of abandoned fields in and around Skoura with an acceptable class accuracy of 74–76% (Fig. 6). Desertification detection accuracy was limited by some confusion with other classes, including mixed pixels consisting of surviving palms scattered across desertified fields, and the similarity between bare and desertified soil. However our product represents a higher resolution map of desertification based on the extent of abandoned fields, when compared to existing historical land degradation products which are generated from hindcasted models. Our workflow revealed that 11 km^2^ of the 175 km^2^ AOI is desertified. The areas of densest cultivation seem to be in the centre of the oasis, and still have at least intermittent input from the surviving khettara. Future work could examine the correlation between the presence of the most reliably-functioning khettara and healthiest areas of cultivation. Desertified fields are concentrated in areas to the north and at the centre of the oasis. The area to the north may have been formerly used for seasonal cultivation fed mainly by diversion from wadis, rather than by the khettara. The cultivated area (treecover and cropland) covers 24 km^2^. The khettara segments and desertified fields were compared to KH9 images from 1971 and 1982. Although much of these former fields had desertified prior to the 1971 image, examination of the GE and KH9 satellite imagery as well as archival reports and the community surveys reveal a dynamic chronology of water management in the Skoura oasis which feed the surviving areas of cropland.

The first official documentation of the khettara in Skoura begins with a program during the French protectorate (1912–1956). Between the 1930s to 1950s, the French involved local residents in the construction of a new khettara system named Makhzen, (“Government”)^60^ and correlates to our Groups 1 and 34–36 (Fig. 6). With the end of the protectorate, regional agricultural authority shifted to the Regional Agricultural Development Office of Ouarzazate (ORMVA). The KH9 imagery indicates that 80% of the currently identified khettara segments were present by at least 1971 (n = 215).

Between 1960 and 1989, the ORMVA completed two major irrigation rehabilitation and modernization plans, including a 1960 project to continue efforts on the Makhzen khettara. KH9 imagery indicates that 12% of the Skoura khettera segments were constructed between 1971 and 1982 (n = 32). By the time the last KH9 satellite image was captured in 1982 a further 8% of new khettara segments were present (n = 32). In 1989, the ORMVA led a project to reinforce the seguia canals and khettara ventilation/maintenance shafts with concrete. During the late 1980s and 1990s, the ORMVA redirected efforts towards installing pump wells as many of the khettara were drying up as a result of drought^60^.

Many of the khettara systems have been modified and restored through recent local initiatives. Ait Kandouch recorded 48 khettaras in 2000, 16 of which were inactive^21^. Several local restorations were completed between 2004 and 2023. One of the most common projects was to reinforce ventilation/maintenance shafts with concrete. Several systems were modified using a “cut-and-cover” method which involves excavating and rebuilding a portion of or the entire system with concrete (Fig. 8d).

In addition to the revitalisation of khettara systems, over the past several decades there have been concerted efforts to protect hydraulic features and cultivated areas. Lateral erosion from seasonal wadis has defined oasis boundaries, cutting or widening areas depending on the meandering flow. Erosional processes have also infilled and eroded khettara, canals, and fields. Wet-lain stone walls, gabion baskets, and earthen dikes are evident primarily along the Oued el Hajjaj. The majority of these works were implemented post-1982, however a series of walls and gabions were constructed between 1982 and 2008 to protect the mother wells of khettara in Groups 2 and 3 with a modification to the original wall in 2022 when revitalisation of a khettara occurred (Fig. 7).

Traditional and indigenous knowledge is now recognised as important in ensuring sustainability^4^, especially in tandem with scientific knowledge^3^. The khettara systems of the Saharan oases rely heavily on the ability to be managed at a local level. Water management depends on community members’ ability to adapt and react to change and communal understanding of water rights. In Morocco, community members own a slot within an irrigation cycle (known as nouba). Families own certain hours of irrigation rights (e.g. 1 h on day 5 of the cycle). The nouba system is usually established when a khettara or canal is first built, and families who assisted in the construction of the system will receive a certain allotment. These rights are then passed down within the family. Water in Morocco is predominantly managed by local associations (Jmâa) that solve issues related to water including raising funds and asking for volunteers to undertake irrigation maintenance.

Historically, water management relied on this communal system to maintain equal distribution. This ensured that upstream oases didn’t divert all water from the three wadis which flow through Skoura oasis. However, communal understandings of water began to shift during the French protectorate and the introduction of modern irrigation technology such as motorised pumps that allow deep groundwater to be accessed significantly faster, and more unsustainably than traditional methods. Pump wells are a major cause of rendering khettara systems inactive; they can lower the aquifer level which will affect the physical structure of the khettara systems, and they are a private source of water and irrigation which disrupts communal agricultural practices^61–65^. By 1996, Skoura had 74 motorized pump wells, and this number has increased significantly over the past two decades^21^.

Significant recent periods of drought occurred during the 1980s and early 2000s; the most recent lasting from 2014 to 2022. During these droughts, many of the khettara systems began to run dry (Fig. 1 shows khettera activity levels identifiable in the field and from satellite imagery). As a result of the 1980s droughts, many of the southern khettara systems in Skoura oasis ceased production. The demise of these systems prompted ORMVA to redirect efforts away from maintaining khettara. However, even though intense periods of drought may affect many khettara, if infrastructure is maintained, water will return when the rains do. Rainfall in 2005 resulted in the resurgence of several “abandoned” khettara systems in Skoura^60^. Seasonal resurgence has been impacted by the current drought in Morocco, which is one of the worst in living memory and has caused further drying up of many khettara^66^. In contrast, the winter and spring of 2023 were one of the wettest in recent years and have begun to positively affect river irrigation and the resurgence of khettara*.* Only two khettara segments were active during an initial site visit of a neighbouring oasis in November–December of 2022. However, during a second visit in February 2023, after heavy rainfall, an additional two khettara systems had become active and were being utilised. The satellite imagery shows that others in Skoura have also been recently in use. If the large areas of historical desertification identified around Skoura were caused by drought in the past, it must have been of long-enough duration that systems were not revitalised.

In Saharan oases like Skoura, traditional irrigation methods are threatened due to climate change and modern pressures on resources. Water scarcity and aridity has already had an impact on Morocco^4^. Areas of abandoned fields represent different phases of desertification caused by multiple factors, but the extent and chronology of these processes is not well represented by the existing land degradation products, which in addition are not all readily available as open-source datasets. As well as making our code open-access, we have used fully open-source data (in this case Copernicus) to ensure both replicability and access to our workflow. Although high-resolution imagery allows smaller details to be detected, Copernicus is committed to remaining free and open-access^67^.

The existing global land degradation products also tend to be inaccurate. They rely on the convergence of several evidence types. The data used can come from several different sources and by making it interoperable, some accuracy may be lost, and error can be propagated. For example, Trends.Earth relies on NDVI derived from MODIS but also the ESA CCI Land Cover dataset which is derived from the ENVISAT MERIS time series^68, 69^ and the SoilGrids map which is derived from machine-learning methods applied to different datasets^70^.

The global products often utilise existing datasets, databases and model outputs, which in themselves are created using series of other, older datasets (for example see Campbell et al.^71^ who used the HYDE and SAGE databases for historical cropland data, the SAGE data in turn used the model of Ramankutty and Foley^38^, who used Houghton and Hackler’s model^72^, who themselves used a series of sources). Identifying the original source of the data involves tracking back through many sequences of modelling and compilation. None of the landcover products available have a class for desertified land, regardless of the date of desertification. A landcover product representing this class could improve the results of the existing models.

Based on their models, Klein Goldewijk et al.^11^ suggest a global irrigated area in 1700 CE of 4.5 Mha, with the extent of irrigated land being small compared to overall agricultural land until the twentieth century. Expansion of our machine-learning based workflow could test this hypothesis for the Sahara. Applied to a case study of Skoura, our model shows that there are significant areas of this oasis which had desertified prior to the 1970s and this is confirmed by the historical satellite imagery (Fig. 7); the historical extent of oasis cultivation in the Sahara may have been more extensive, and also more dynamic than currently recognised. Desertification is likely to have occurred over several phases; proxy records reveal drier conditions since the 1970s but also historically^73, 74^. Dry conditions in Morocco in the 10th–fourteenth centuries AD could have led societies to invest in irrigation as an adaptation strategy, as has been suggested for the Tafilalt, but this cultivated extent later contracted. Similarly, there was investment in irrigated cultivation in the Draa in the eleventh-thirteenth centuries AD^75^. This was followed by desertification of areas of the oasis in the sixteenth century, possibly due to changing dynamics of the river from which the canal systems abstracted^75^.

## Conclusion

One of the common misconceptions regarding traditional oases is that khettara and canal systems were only constructed in antiquity, and communities have been simply maintaining them. However, archaeological, archival, and anthropological research points to a more complex story. For the past 100 years, these systems’ construction, use, and abandonment have been dynamic. In Skoura khettara systems have experienced numerous waves of development, reconstruction, and patterns of use, disuse, and revitalisation.

A comprehensive understanding of the long-term processes that led to the earlier phases of desertification mapped using our workflow is needed. Optically Stimulated Luminescence (OSL) dating^76^ could be used to further refine the chronology of cycles of cultivation and desertification in both Skoura and the wider Sahara. This includes the earliest phases of irrigated cultivation and use of technologies such as khettara. Skoura may be comparable to the Tafilalt, where irrigation was originally based on diverting surface water using canals, with khettara used after the fourteenth century AD^77^.

Existing landcover classification products form a key input to models of land degradation and historic cropland extent but do not identify degraded land as a distinct class. Incorporating an empirical desertification classification dataset into these models, such as the one presented in our paper, contextualised by historical and archaeological evidence, could improve their accuracy and value. The need for accurate historic land cover maps to be used in climate change modelling, including future scenario models, is recognised^11^. Our analysis reveals that the croplands of Skoura shrunk by around 11 km^2^ prior to the 1970s, and that some of this land may have been irrigated by diversion of surface water. It's possible that the modern extent of Saharan oases is less than that of its historical extent. In the face of current challenges posed by climate change and over-exploitation, the problems that lead to such fragility urgently need investigation.

### Supplementary Information

Supplementary Information 1.

Supplementary Information 2.

Supplementary Legends.

## Data Availability

The datasets generated during the current study can be downloaded by running the JavaScript and/or Python code from a publicly available repository here https://figshare.com/s/b4f8a11064783ea39395. The primary input datasets and parameters used for the algorithm are delineated in the code. The khettara dataset is available as a shapefile and in GeoJSON format in our supplementary information, available here https://figshare.com/s/fb9a9f0ae3a0935af252.

## Code availability

The desertification detection code can be accessed here https://figshare.com/s/b4f8a11064783ea39395. The code can be executed by signing up for a free Google Earth Engine account. By running this code, the same datasets can be generated. The inputs selected and filter parameters can also be identified by viewing the code.
