# Supplementary figures and images for: Detecting desertification in the ancient oases of southern Morocco
Source: Sci Rep. 2023 Nov 8;13:19424. doi: 10.1038/s41598-023-46319-1 (PMC10632388; doi:10.1038/s41598-023-46319-1)

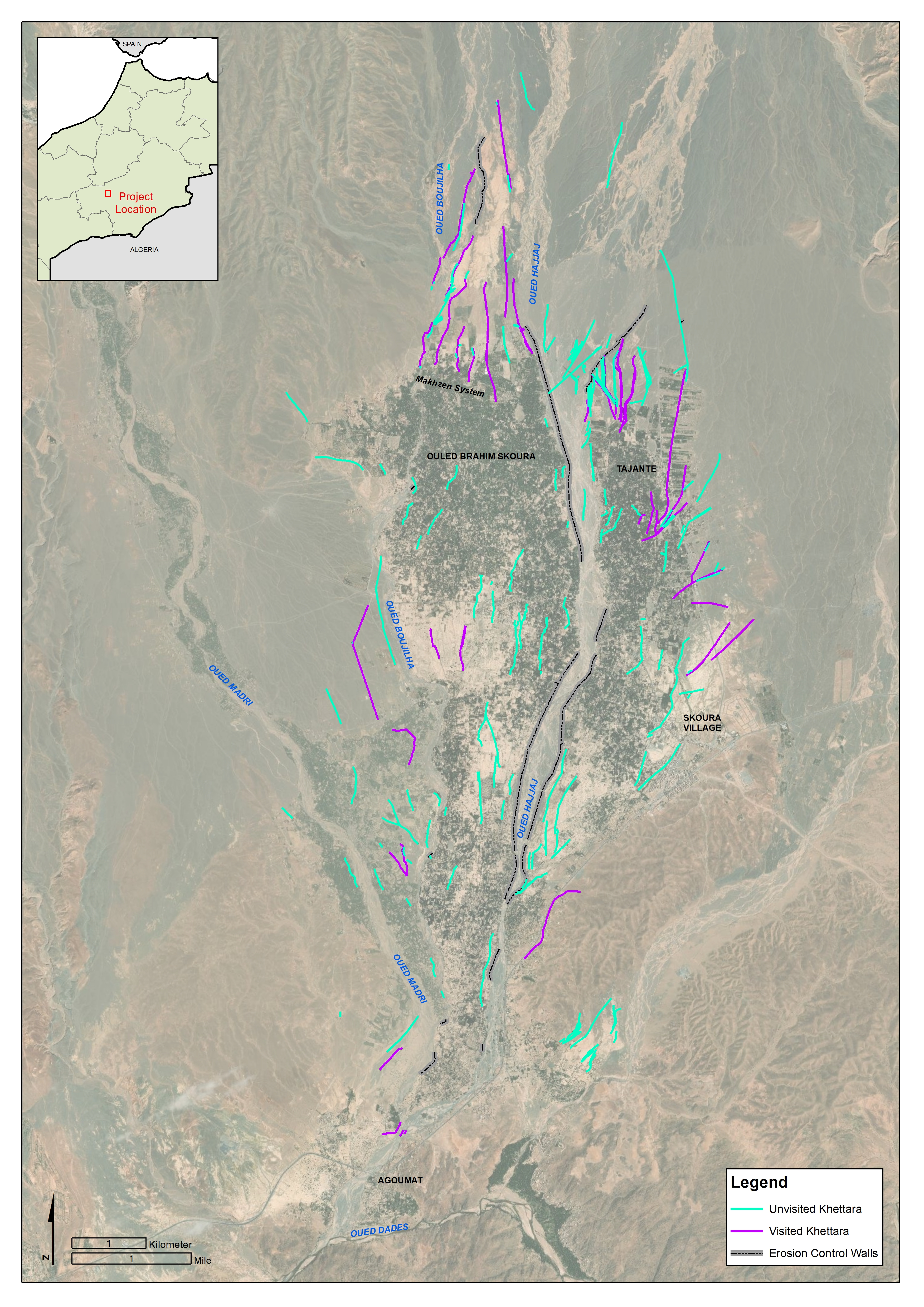

Supplement: Supplementary file 1 — Supplementary Information 1. [file 41598_2023_46319_MOESM1_ESM.zip › Data/FigS1_Visited_Khet.jpg]
