# Supplementary material for: Detecting desertification in the ancient oases of southern Morocco
Source: Sci Rep. 2023 Nov 8;13:19424. doi: 10.1038/s41598-023-46319-1 (PMC10632388; doi:10.1038/s41598-023-46319-1)
Supplement: Supplementary file 3 — Supplementary Legends. [file 41598_2023_46319_MOESM3_ESM.docx]

# **Supplementary Information**

Supplementary data S1 Shapefile and geojson of khettara

Fig S1 Map of visited and unvisited khettara

Table S1 Image Interpretation Key

Table S2 Khettara group summary
